# Prediction and optimization of epoxy adhesive strength from a small dataset through active learning

**DOI:** 10.1080/14686996.2019.1673670

**Published:** 2019-10-02

**Authors:** Sirawit Pruksawan, Guillaume Lambard, Sadaki Samitsu, Keitaro Sodeyama, Masanobu Naito

**Affiliations:** a Data-driven Polymer Design Group, Research and Services Division of Materials Data and Integrated System (MaDIS), National Institute for Materials Science (NIMS), Tsukuba, Japan; b Program in Materials Science and Engineering, Graduate School of Pure and Applied Sciences, University of Tsukuba, Tsukuba, Japan; c Energy Materials Design Group, Research and Services Division of Materials Data and Integrated System (MaDIS), National Institute for Materials Science (NIMS), Tsukuba, Japan; d Department of Advanced Materials Science, Graduate School of Frontier Sciences, The University of Tokyo, Kashiwa, Japan

**Keywords:** Materials informatics, active learning, adhesive joint strength, epoxy resin, crosslink network structure, 600

## Abstract

Machine learning is emerging as a powerful tool for the discovery of novel high-performance functional materials. However, experimental datasets in the polymer-science field are typically limited and they are expensive to build. Their size (< 100 samples) limits the development of chemical intuition from experimentalists, as it constrains the use of machine-learning algorithms for extracting relevant information. We tackle this issue to predict and optimize adhesive materials by combining laboratory experimental design, an active learning pipeline and Bayesian optimization. We start from an initial dataset of 32 adhesive samples that were prepared from various molecular-weight bisphenol A-based epoxy resins and polyetheramine curing agents, mixing ratios and curing temperatures, and our data-driven method allows us to propose an optimal preparation of an adhesive material with a very high adhesive joint strength measured at 35.8 ± 1.1 MPa after three active learning cycles (five proposed preparations per cycle). A Gradient boosting machine learning model was used for the successive prediction of the adhesive joint strength in the active learning pipeline, and the model achieved a respectable accuracy with a coefficient of determination, root mean square error and mean absolute error of 0.85, 4.0 MPa and 3.0 MPa, respectively. This study demonstrates the important impact of active learning to accelerate the design and development of tailored highly functional materials from very small datasets.

## Graphical abstract

**Figure:**
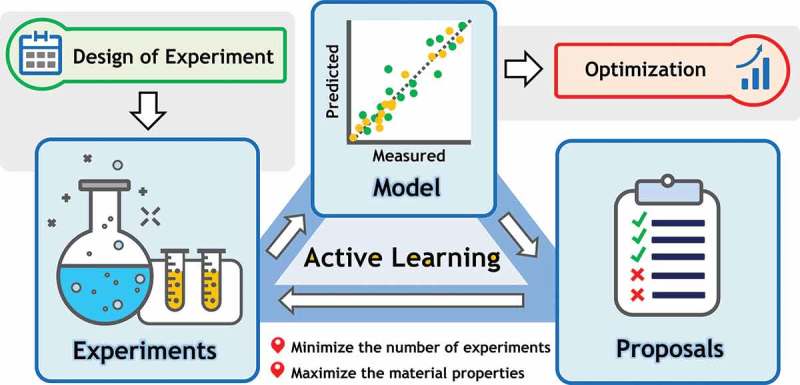

## 1. Introduction

In recent decades, interest in machine-learning (ML) techniques has increased in various research fields because of their outstanding efficiency to extract salient information [1]. More recently in the field of materials science, ML techniques have begun to play an important role in the design and development of novel materials [2,3]. ML usually requires a large amount of data, that is, > 1000 samples, to build accurate models [1]. The main goal of ML in materials science is to search for highly functional materials with properties that are tailored to fit the requirements of a specific application [2]. Recent studies demonstrate the potential of ML-based experimental design to discover various new functional materials in different fields within an active learning framework. The active learning strategy is typically efficient in improving prediction model. The examples of this include finding very low thermal hysteresis NiTi-based shape memory alloys using adaptive experimental design [4], discovery of large electrostrains in BaTiO_3_-based piezoelectrics using active learning [5], searching high-temperature ferroelectric perovskites by two-step machine learning [6], finding BaTiO_3_-based ceramics with large energy storage at low fields using machine learning and experimental design [7] and discovery of new metallic glasses through iteration of machine learning and high-throughput experiments [8]. However, a ML based approach has not been widely applied to the field of polymer science. One major constraint is that experimental datasets in polymer science are typically limited and expensive to construct. A huge and comprehensive source of information on polymer properties is not easily obtainable. Sometimes, the experimental dataset is scattered [2]. Datasets that are collected from various literature sources may be noisy and inconsistent because several experimental factors affect any obtained sample and measurements, such as process conditions, the source and purity of used chemicals and environmental conditions [9,10]. Particularly, if a material requires a specific design, only few data are available. Thus, it is challenging to obtain a sufficiently large curated dataset, which limits the use of ML for polymer research.

The development of high-strength adhesives for joint bonding is one of the cases where application-specific design is needed. In addition to adhesive properties, several other factors influence the adhesive joint strength (σ_ad_), such as: substrate properties, substrate surface preparation, joint configuration, measurement conditions and environmental factors. Hence, an adhesive will behave differently under different joint-design and bonding conditions [11]. In consequence, the experimental dataset for an adhesive for one specific joint cannot be acquired easily because different studies usually use different conditions, such as an adhesive thickness, substrate surface treatment and the joint configuration. Furthermore, no theoretical and empirical knowledge exists to predict precisely the σ_ad_ from a modified adhesive system.

Various approaches have been exploited in the literature to modify the mechanical properties of adhesives, such as the fracture toughness, elastic modulus and tensile strength [12–15]. The modification of epoxy adhesives by adjusting their network structure is one of the most effective ways to provide a wide diversity of mechanical properties. Using this approach, we can tailor the adhesive properties to meet a specific requirement for joint bonding. In the case of adhesively bonded joints, that is, when two substrates are bonded via an adhesive, several properties are required to achieve a high σ_ad_. A good resistance to crack growth as reflected by a high fracture toughness and high flexibility of adhesives is a desirable property to withstand the tensile stress concentration of joints [11]. The adhesive needs a reasonably high elastic modulus to obtain a high-shear fracture stress [11]. The interaction between an adhesive and the substrates is important for controlling the fracture behaviour of joints [16]. Because several factors influence adhesively bonded joint properties, the development of high-performance adhesives for joint bonding is more complicated than that for the bulk form, and requires further advanced techniques for achieving an exceptionally high σ_ad_ [11,17]. In the metal joining process, especially in structural bonding, an adhesive with high σ_ad_ is highly desired to resist joint failure and impact forces [18].

Therefore, we propose a combination of the design of experimental techniques with an active learning (as known as optimal experimental design [19]) pipeline and a Bayesian optimization to model and maximize the σ_ad_ from various mixtures to overcome the issues presented above. Compared to other machine-learning-based materials’ design approaches [4–8], our two-stage data-driven approach allows us to propose an optimal condition for achieving target property from a very small dataset with designing controlled experiments, and does not require data from previous literatures. The first stage, active learning, aims to construct an accurate ML model with a particular focus only on a specific range of high σ_ad_. By refining the experimental conditions in the second stage, the Bayesian optimization is refined to search for the adhesive materials with extremely high adhesive strength. This approach is foreseen to accelerate materials design and reduce the development cost and time, especially for which initial number of samples is limited compared to the number of combinations of free parameters for their formulations.

We use an initial small experimental dataset that we built and controlled. This dataset is focused on a model adhesive system that is composed of conventional bisphenol A-based epoxy resin and an amine-terminated poly(propylene glycols) curing agent that is described in Section 2.1. The use of these types of epoxy resins and curing agents with different linear chain lengths allows us to tailor the adhesive properties. Throughout this paper, σ_ad_ is measured through a single-lap shear test presented in Section 2.2. To obtain epoxy adhesives with various network structures, 32 samples of epoxy adhesives were prepared from different epoxy resin molecular weights (MW_E_), curing agent molecular weights (MW_C_), amine-to-epoxide ratios (r) and curing temperatures (T_cure_) according to conditions suggested by a Graeco–Latin square design as shown in Section 2.3. The experimental results are reported in Table S2 of the supplemental materials and they are used as our initial curated dataset. Then, various ML models were trained on this dataset to predict the σ_ad_. To enhance the prediction accuracy of the most promising ML model and to increase the dataset size (n_s_) iteratively, an active learning pipeline was applied as detailed in Section 2.4. Therefore, specifically targeted experiments for reaching a high σ_ad_ were conducted. After achieving experimental-like accuracy on σ_ad_ predictions, the obtained ML model was fixed. Finally, a Bayesian optimization was used to optimize an epoxy network structure in greater processing detail and achieve the reported extreme high σ_ad_ in this study. Indeed, the Bayesian optimization highly depends on its forward ML model for making proposals. Then, avoiding the active learning step would be equivalent to reduce the Bayesian optimization to a naive random sampling of our features space. This kind of strategy is here proposed in the case that the initial dataset is very small, which is often found in the field of polymer science. We present the promising results in Sections 3.1 and 3.2 to accelerate the discovery of new application-specific materials by using a very small experimental dataset (few tens of samples). An understanding of those predictions, as discussed in Section 3.3, should provide valuable knowledge for the future development of adhesive materials. Finally, we conclude and discuss further possible improvements in Section 4.

## 2. Experimental and ML methods

### 2.1. Materials

**Figure 1.**
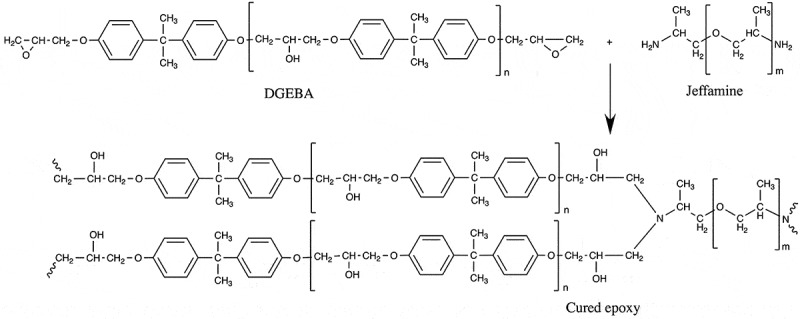
Chemical structures of diglycidyl ether of bisphenol A-based epoxy resin (DGEBA) and amine-terminated poly(propylene glycols) curing agent (Jeffamine) and their curing reaction.

Diglycidyl ether of bisphenol A-based epoxy resin (DGEBA) and amine-terminated poly(propylene glycol) curing agent (Jeffamine) with four different molecular weights were used: MW_E_ ∈ {370, 1650, 2900, 3800} g/mol for the DGEBA (Mitsubishi Chemical, Japan) and MW_C_ ∈ {230, 400, 2000, 4000} g/mol for the Jeffamine (Sigma-Aldrich, Japan). The chemical structures of the DGEBA and Jeffamine are shown in Figure 1. All chemicals were used as received without further purification. Aluminium alloy A6061P-T6 (100 mm × 25 mm × 2 mm) was used as a substrate. Prior to the adhesive joint fabrication, the substrate surfaces were sandblasted and cleaned with ethanol and acetone.

### 2.2. Preparation of adhesive joint specimens and single-lap shear test

**Figure 2.**
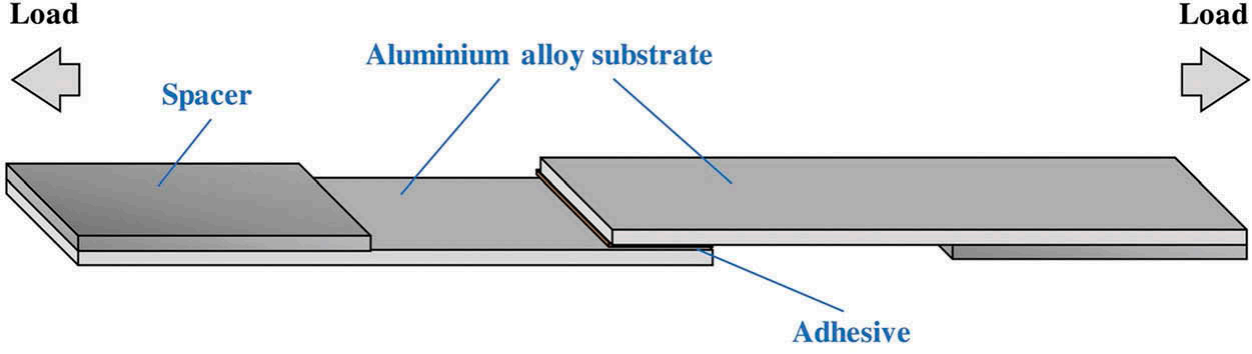
Schematic illustration of adhesive joint specimen for single-lap shear test.

A DGEBA epoxy resin (5.0 g) was preheated at 190°C for 30 min to melt crystals. The Jeffamine curing agent was added to the liquid epoxy resin at a specific ratio r ∈ {0.75, 1.0, 1.25, 1.5}, where r < 1.0 indicates an epoxy excess, r = 1.0 indicates a stoichiometric mixture between the amine and epoxide and r > 1.0 indicates an amine excess. For example, an r of 1.25 means 25% excess amine. The epoxy resin and curing agent were mixed by hand at 190°C for a few seconds to achieve a homogeneous blend. This adhesive precursor was spread over a 25 mm × 12.5 mm area on one face of a pair of substrates. The two substrates were bonded together and the overlapping area was fixed by metal clamps as described previously [20]. An illustration of the adhesive joint specimen is provided in Figure 2. The prepared specimen was cured in an oven at a specific temperature T_cure_ ∈ {90, 130, 170, 210}°C for one hour. The adhesive thickness was maintained at ~100 μm using 0.1 parts per hundred resin of spherical glass bread (Fujiseisakujo, Japan) as spacers. The four variable parameters used later as input features for the ML models (see Section 2.4.2) are summarized in Table 1. The parameter values in Table 1 are typical values of MW_E_, MW_C_, r and T_cure_ for adhesive preparation. To be specific, MW_E_ and MW_C_ were selected on the basis of commercially available source material, and the values of r and T_cure_ were chosen within a range that allow sample preparation.

**Table 1.** Summary of variable parameters for adhesive formulation used at the active learning stage. Variable parameters include the molecular weight of the epoxy resin MW_E_ (g/mol), the molecular weight of the curing agent MW_C_ (g/mol), the amine-to-epoxide ratio r and the curing temperature T_cure_ (°C).

	Variable parameter			
No	MW_E_ (g/mol)	MW_C_ (g/mol)	r	T_cure_ (°C)
1	370	230	0.75	90
2	1650	400	1	130
3	2900	2000	1.25	170
4	3800	4000	1.5	210

The single-lap shear test of the adhesive joint specimen was carried out by using a 10-kN AG-X plus series universal tensile testing machine (Shimadzu, Japan). All tests were performed at a 2-mm/min crosshead speed at room temperature. The σ_ad_ was calculated by dividing the maximum tension load by the area of overlap (25 mm × 12.5 mm). At least two specimens were used for each measurement and the average value was reported with the standard deviation. Indeed, the maximum tension load that was reached by the developed epoxy resin of the highest σ_ad_ exceeded 10 kN. Therefore, a second 50-kN AG-X plus series universal tensile testing machine (Shimadzu, Japan) was needed at the final stage of our design study. The use of this second machine was required only when we had reached the measurement limitation of the first one.

### 2.3. Selection of experimental conditions for the initial dataset

The experimental conditions in this study consisted of 256 possible conditions that were provided by a combination of four molecular weights for the epoxy resin and the curing agent, four amine-to-epoxide ratios and four T_cure_ values (see Table 1). An initial set of n_s_ = 32 samples was collected according to the conditions that were suggested by a Graeco–Latin square design [21]. The Graeco–Latin square design is a design of experimental techniques that can generate a uniform sample of scattered data points [22]. By conducting two replicated four-by-four Graeco–Latin square designs, 32 experimental conditions were obtained.

### 2.4. ML method

Data pre-processing, data splitting and the application of the ML algorithms was performed using the Python package Scikit-learn (version 0.21) [23], and the Bayesian optimization was executed using the Python package GPyOpt [24].

#### 2.4.1. Data pre-processing and splitting

The four variable parameters in this study (see Table 1) were standardized following a standard Gaussian distribution of mean zero and standard deviation of one [17]. A k-fold cross-validation of different ML algorithms was performed [25]. The dataset was split randomly into k folds of equal size. Each fold was used as a training set by an ML algorithm with one other fold kept as a test set. The process was repeated k times. Their mean absolute error (MAE), root mean square error (RMSE) and coefficient of determination (R^2^) of the property predictions versus observations were averaged across all k folds to evaluate the ML models. When a validation set was required for early stopping (e.g. for Gradient boosting), the training set was split so that 80% of the original training set was retained for training and 20% was used for validation.

#### 2.4.2. ML algorithms

Three supervised ML algorithms were applied as a regression tool to our dataset: Elastic Net, Random forest and Gradient boosting [23]. Elastic Net is a linear regression model, whereas Random forest and Gradient boosting are ensemble learning methods that make predictions by combining the outputs from individual regression trees. The Random forest builds each regression tree independently and merges them to obtain accurate and stable predictions, and Gradient boosting builds regression trees sequentially to minimize residual errors from the previous trees. XGboost in Scikit-learn library was used to train Gradient boosting model [23]. During Gradient boosting training, early stoppage was applied to minimize the overfit on the training set [26]. The accuracy of an ML model was accessed through their RMSE (a lower value is better), MAE (a lower value is better) and R^2^ (a value closer to one is better) on the predictions versus observations via a k-fold cross-validation.

#### 2.4.3. ML model and active learning

**Figure 3.**
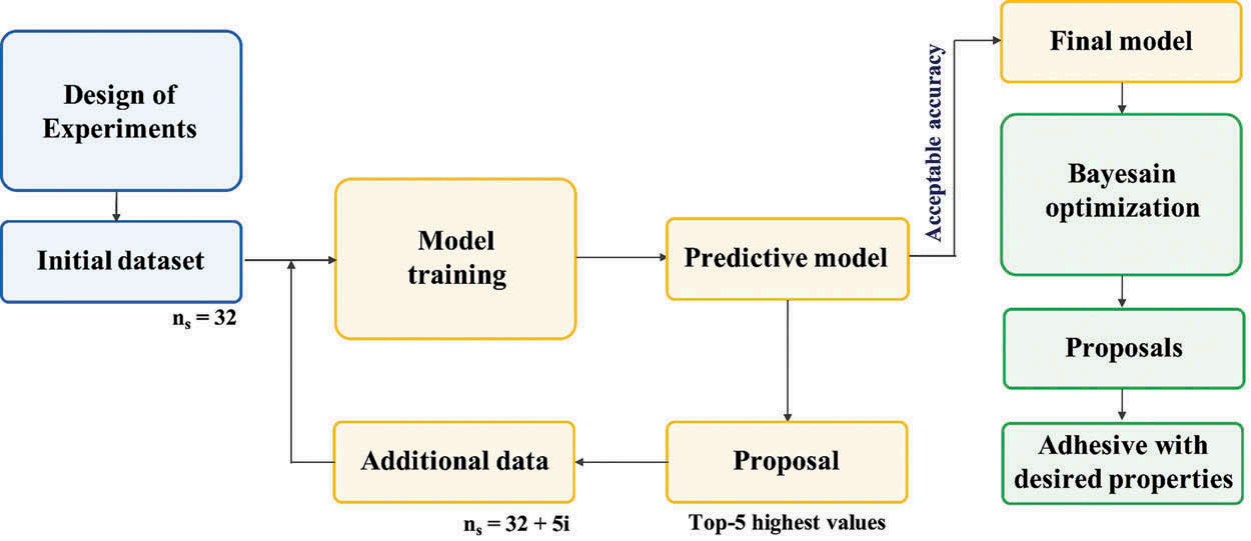
Flowchart of our proposed approach for modelling and optimization. Note that n_s_ indicates the dataset size and i indicates the number of cycles.

The best ML model that was chosen for its accuracy to predict the σ_ad_ was trained on the initial dataset of n_s_ = 32 samples. The model predicted the σ_ad_ of all (256–32) possible experimental conditions (see Table 1) from the initial dataset. The predicted σ_ad_ were ranked in descending order. The top-five ranked experimental conditions were selected as proposals for the next measurements to be performed in the laboratory to increase the σ_ad_. These new measurements were added to the initial dataset of now n_s_ = (32 + 5) samples. Then, the ML model for σ_ad_ prediction was trained again on this improved dataset. The ML model improved its σ_ad_ prediction with additional data, especially for a range of high σ_ad_, and proposed again the experimental conditions to follow for the next measurements. This type of iterative supervised learning, or so-called active learning, was repeated cycle after cycle until a preliminary goal of a sufficiently high accuracy of the ML model was reached. In this study, active learning was stopped if the prediction error was comparable to the experimental error of the σ_ad_ that was measured by a single-lap shear test. The final ML model was kept fixed and used as a forward model for a subsequent Bayesian optimization. The available experimental data at this stage of active learning were fed to the Bayesian optimization as initial data points. The flowchart of the active learning method is shown in Figure 3. Compared to conventional ML approaches, we use an initial experimental dataset that we built and controlled by design of experiments techniques. This technique would generate a highly uniform set of sample points (Figure S1). In addition, all of the sample preparation and measurements is carried out under the same experimental environment resulting in accurate and consistent data.

#### 2.4.4. Bayesian optimization

A Bayesian optimization [27] was used to search for the highest σ_ad_ by refining the variable conditions from Table 1 once the coarse optimization through active learning had been terminated. The Expected Improvement (EI) was used as an acquisition function to propose new experimental conditions to maximize the σ_ad_. In this step, two experimental conditions were refined: r and T_cure_. The r could vary from 0.75 to 1.50 with an increment of 0.01, and the T_cure_ could vary from 90 to 210°C by an increment of 1°C. The MW_E_ and MW_C_ were kept as four possible discrete values because these are difficult to control precisely. Thus, the proposed experimental conditions from the Bayesian optimization were ranked in descending order with respect to the predicted σ_ad_. A series of experiments was carried out starting from rank 1 until a new highest σ_ad_ was observed.

## 3. Results and discussions

### 3.1. Experimental results from the initial dataset

**Figure 4.**
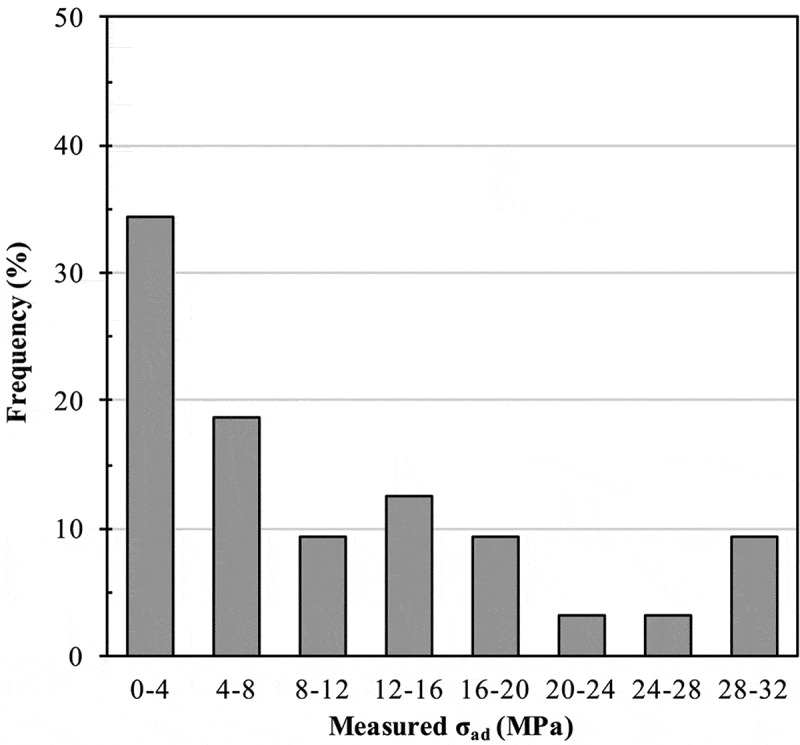
Distribution of adhesive joint strength σ_ad_ (MPa) from the initial dataset of size n_s_ = 32 samples.

Experimental measurements of σ_ad_ that compose our initial curated dataset are reported in Table S2 of the supplemental materials. Figure 4 shows the distribution of σ_ad_ experimental values. σ_ad_ was distributed from 0.0 MPa (no bond strength) to 31.9 MPa with an average at 10 ± 9 MPa.

### 3.2. ML model

#### 3.2.1. Assessment and selection of an σ_ad_ prediction model

**Figure 5.**
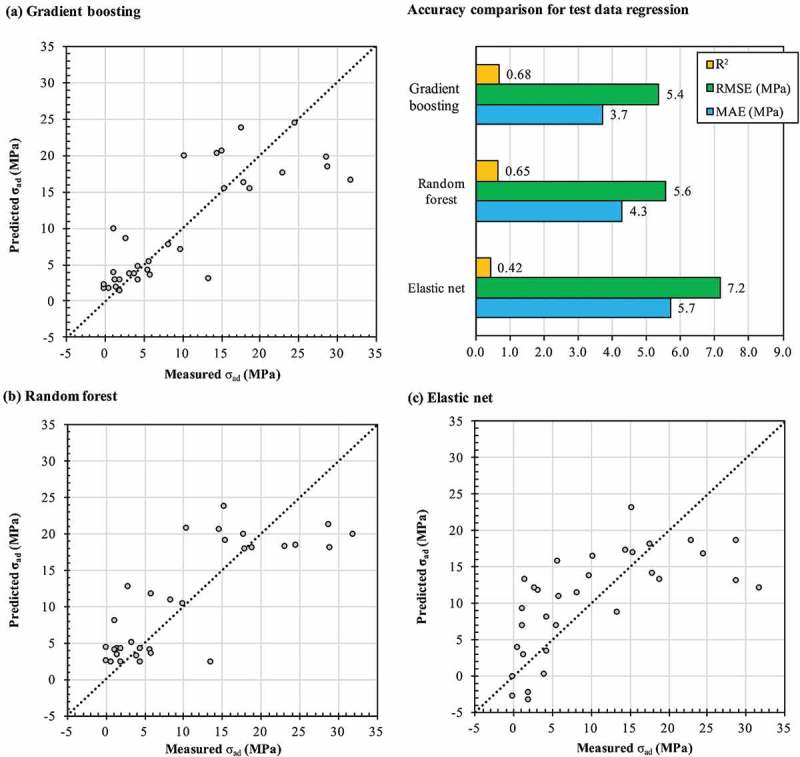
Distribution of predicted versus measured adhesive joint strength σ_ad_ (MPa) from successive test sets used in the 32-fold cross-validation using different ML algorithms: (a) Gradient boosting, (b) Random forest and (c) Elastic Net. A dashed straight line indicates equal measured and predicted σ_ad_. Hyperparameters used for these runs are shown in Table S4 of the Supplemental material.

Gradient boosting, Random forest and Elastic Net performance were checked through a 32-fold cross-validation. The comparison of predicted against measured σ_ad_ for each algorithm is shown in Figure 5. A dashed straight line indicates an exact match between the predicted and measured values. The Random forest and Gradient boosting algorithms could capture non-linear relationships among the variable parameters that cannot be accessed via a linear regressive model, such as Elastic Net. Their indicated RMSE and MAE in Figure 5 were averaged over the 32 folds, and the R^2^ was calculated to evaluate their prediction accuracy. A comparison of the accuracy for each algorithm is shown in Figure 5 (top-right). The Elastic Net model showed the lowest accuracy of R^2^, RMSE and MAE, and therefore, was discarded. The Gradient boosting model showed a slightly better accuracy than the Random forest model in terms of a higher R^2^ value, and lower RMSE and MAE values. Hence, the Gradient boosting algorithm was selected to predict the σ_ad_ in further steps.

#### 3.2.2. Active learning and ML model performance

**Figure 6.**
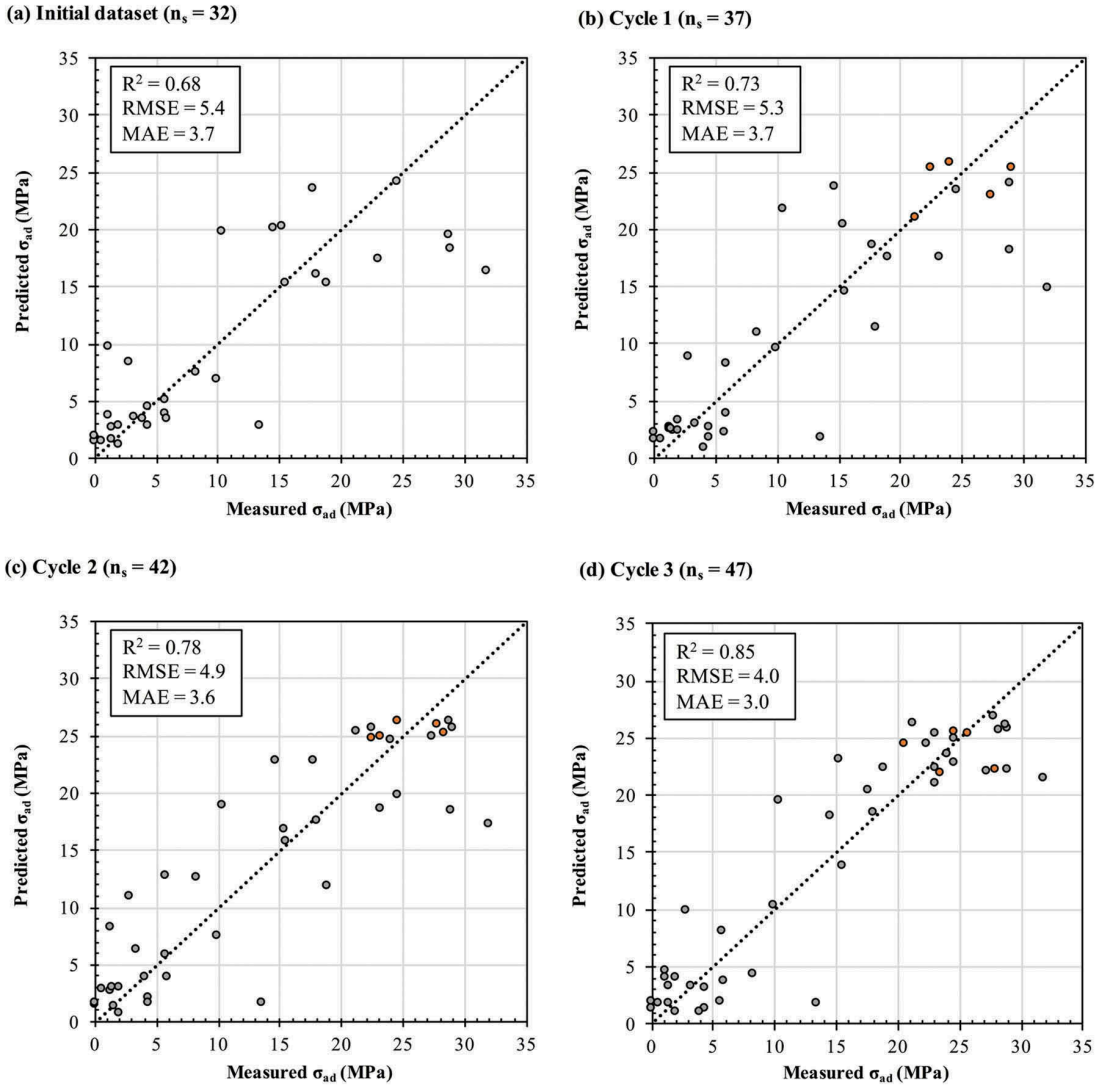
Correlation scatter plots (test data) of predicted and measured adhesive joint strength σ_ad_ (MPa) using different dataset sizes n_s_ (samples): (a) initial dataset, (b) cycle 1, (c) cycle 2 and (d) cycle 3. Grey and orange dots indicate data from existing and new measurements, respectively, at cycle i. All proposed experimental conditions are summarized in Table 2. Hyperparameters used for these runs are shown in Table S4 of the Supplemental material.

In Section 3.2.1, the Gradient boosting model was selected to predict the σ_ad_ based on different experimental conditions. The σ_ad_ of all remaining (256–32) possible experimental conditions were predicted and ranked in descending order. The top-5 experimental conditions with the highest σ_ad_ were proposed for measurements. The new measurements were re-used in the Gradient boosting model to improve the accuracy. This process from the prediction phase to the re-injection phase summarizes one cycle of the active learning pipeline. Table 2 lists the top-five proposed experiments for each three cycles of active learning with the corresponding predicted and measured σ_ad_. The measured σ_ad_ in Table 2 that are above ~20 MPa show that the Gradient boosting model allows us to classify experimental conditions with a potentially high outcome compared with the others. These additional data of high strength adhesives are very beneficial to further maximization with Bayesian optimization. Without this strategy, the use of Bayesian optimization on the initial dataset with the model in Figure 6(a) would outcome less relevant proposals and wouldn’t be beneficial compared to a simple random sampling. In addition, 90% of proposed experiments require a MW_C_ of ~400 g/mol, a high T_cure_ of 170 and 210°C, and an excess of amine (r > 1), when the MW_E_ can evolve widely across its specific range (see Table 1). Therefore, a high σ_ad_ can be achieved regardless of the MW_E_. However, it is premature to make any further conclusion about optimal adhesive preparations before the r and T_cure_ parameters are relaxed in Section 3.3.

**Table 2.** Proposed experimental conditions during the active learning stage via Gradient boosting with related experimental results. Predicted adhesive joint strength σ_ad_ (MPa) was calculated by averaging the predictions over the 32 folds via cross-validation. Hyperparameters used for these runs are shown in Table S4 of the Supplemental material.

		Proposed experimental condition					
Cycle	Rank	MW_E_ (g/mol)	MW_C_ (g/mol)	r	T_cure_ (°C)	Predicted σ_ad_ (MPa)	Measured σ_ad_ (MPa)
Initial dataset (n_s_ = 32 samples)	1	2900	400	1.00	210	25.6 ± 0.9	24.0 ± 1.1
	2	3800	400	1.00	210	25.5 ± 1.4	21.2 ± 1.2
	3	370	400	1.00	210	25.4 ± 1.2	29.0 ± 0.1
	4	1650	400	1.00	170	25.4 ± 1.2	22.4 ± 1.7
	5	1650	400	1.00	210	25.4 ± 1.2	27.3 ± 1.6
1 (n_s_ = 37 samples)	1	370	400	1.25	210	25.4 ± 1.1	27.8 ± 0.5
	2	370	400	1.25	170	25.3 ± 1.1	28.3 ± 0.9
	3	370	400	1.50	210	25.1 ± 1.9	23.1 ± 0.4
	4	370	400	1.50	170	25.0 ± 1.9	22.4 ± 1.8
	5	1650	400	1.25	210	24.9 ± 0.5	24.6 ± 0.0
2 (n_s_ = 42 samples)	1	2900	400	1.00	170	23.9 ± 0.4	20.5 ± 3.5
	2	370	230	1.00	210	23.7 ± 1.1	24.6 ± 2.0
	3	370	230	1.00	170	23.7 ± 1.1	27.9 ± 0.2
	4	1650	400	1.25	170	23.5 ± 1.4	23.5 ± 1.0
	5	2900	400	1.25	210	23.4 ± 1.1	25.7 ± 0.9

**Figure 7.**
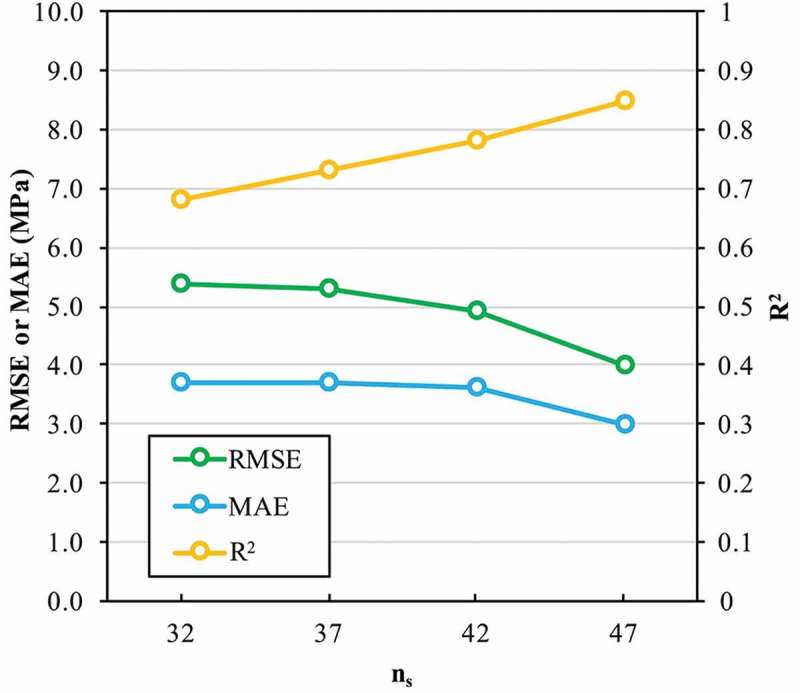
Comparison of the accuracy of the Gradient boosting model to predict the adhesive joint strength σ_ad_ (MPa) for different dataset sizes n_s_ of the dataset.

To show the improvement in accuracy of the Gradient boosting model along the cycles of active learning, Figure 6 presents scatter plots of the predicted versus measured σ_ad_ from the initial dataset to the last cycle. Grey and orange dots indicate existing and new measurements, respectively, at each cycle. As expected, an increase in the dataset size improves the correspondence between the predicted and measured σ_ad_ as summarized in Figure 7 for the corresponding R^2^, RMSE and MAE for the predictions of the σ_ad_ at each cycle beginning with the initial dataset. The R^2^ increases, and the RMSE and MAE decrease gradually with an increase in n_s_. For a dataset of 47 samples, the Gradient boosting model reaches an R^2^, RMSE and MAE of 0.85, 4.0 MPa and 3.0 MPa, respectively. An improvement of 25%, ~26% and ~19%, respectively, was achieved compared with the Gradient boosting model that had trained only on the initial dataset. At cycle three of this active learning pipeline, the prediction performance of the Gradient boosting model became comparable with the maximum standard deviation from experiments (3.5 MPa). Therefore, the active learning procedure was stopped at this stage and the Gradient boosting model was kept fixed based on existing data.

#### 3.2.3. Bayesian optimization

At the Bayesian optimization stage (see Section 2.4.4), the MW_E_ and MW_C_ were kept fixed at the four different values used in Table 1, whereas the r and T_cure_ were varied in steps of 0.01 and 1°C, respectively. The suggested experimental conditions with the highest expected improvement from Bayesian optimization were selected, and a series of experiments was conducted starting from ranking number 1 (Table 3). The new highest σ_ad_ of 35.8 MPa was observed. The σ_ad_ value was considered as a very high σ_ad_ compared with previous studies on epoxy-aluminium joints, which reported a typical σ_ad_ range from ~10 MPa up to 25 MPa [11,28]. Furthermore, this σ_ad_ value was comparable to the commercial epoxy adhesives like Huntsman Araldite 2000+ (26 MPa) and 3M Scotch-Weld DP420 (31 MPa) [29,30], characterized by single-lap shear test. For this sample, the 50-kN tensile machine was used to measure the σ_ad_ because the sample did not break under a 10-kN applied force, i.e. the failure stress of the adhesive joint exceeded the maximum capacity of a 10-kN tensile machine. The suggested experimental conditions from Bayesian optimization showed that a low MW_E_ and a high T_cure_ were a promising condition to reach a high σ_ad_. The MW_C_ and r should be in the middle of their defined range (see Table 1). The σ_ad_ improved for the sample that was prepared with a slight excess of epoxide because other conditions (MW_E_, MW_C_ and T_cure_) in the samples shown in Table 3 were only slightly different. This large improvement in σ_ad_ indicates the suitable balance between strength and flexibility of adhesives [31]. Because excess epoxide (lower r than the stoichiometric ratio) leads to a higher tensile strength but a lower flexibility of adhesives [14], an optimum combination of high strength and good flexibility would be achieved by adjusting the r precisely through Bayesian optimization.

**Table 3.** Proposed preparations of an epoxy adhesive at Bayesian optimization stage with the related experimental adhesive joint strength σ_ad_ (MPa).

	Suggested experimental conditions					
Rank	MW_E_ (g/mol)	MW_C_ (g/mol)	r	T_cure_ (°C)	Predicted σ_ad_ (MPa)	Measured σ_ad_ (MPa)
1	370	400	1.11	199	26.9	28.0 ± 0.7
2	370	400	1.24	194	26.9	27.4 ± 1.2
3	370	400	1.30	191	26.9	18.8 ± 1.4
4	370	400	0.89	209	26.9	35.8 ± 1.1

**Figure 8.**
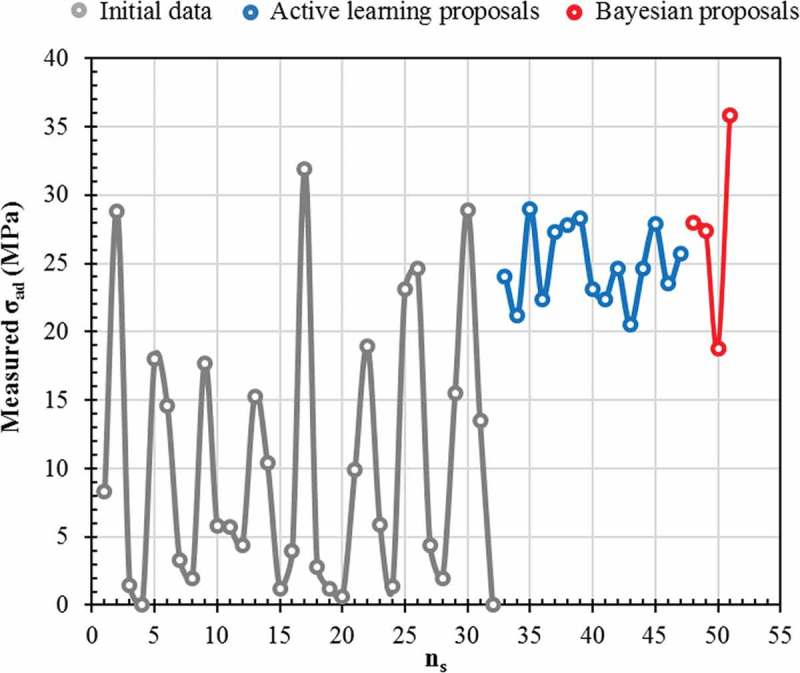
Distribution of adhesive joint strength σ_ad_ (MPa) measurements from the initial dataset alone (grey), after active learning cycles (blue) and after Bayesian optimization (red). Lines are used to guide the eye only.

In summary, Figure 8 illustrates the distribution of σ_ad_ from the initial dataset alone (grey), after three active learning cycles (blue), and after a Bayesian optimization (red). The values of σ_ad_ from the initial dataset were spread randomly from 0 to 31.9 MPa. In contrast, all samples that followed an active learning cycle exhibited a high value of σ_ad_ (> 20 MPa), and one sample from the Bayesian optimization dataset showed an exceptionally high σ_ad_. The spread in measurements from the Bayesian optimization was wider than that from the active learning cycles. A Bayesian optimization balances the exploitation (surrogate model predicts a high objective) and exploration (sampling of regions where the prediction uncertainty is high) of the epoxy adhesive preparation parameters space, where our active learning pipeline based on the ML model predictions only exploits the parameters. These results demonstrate the potential of our method for the design and development of new functional materials when the initial number of samples is reduced compared with the number of combinations of free parameters involved.

### 3.3. Interpretation of ML model for adhesive design

**Figure 9.**
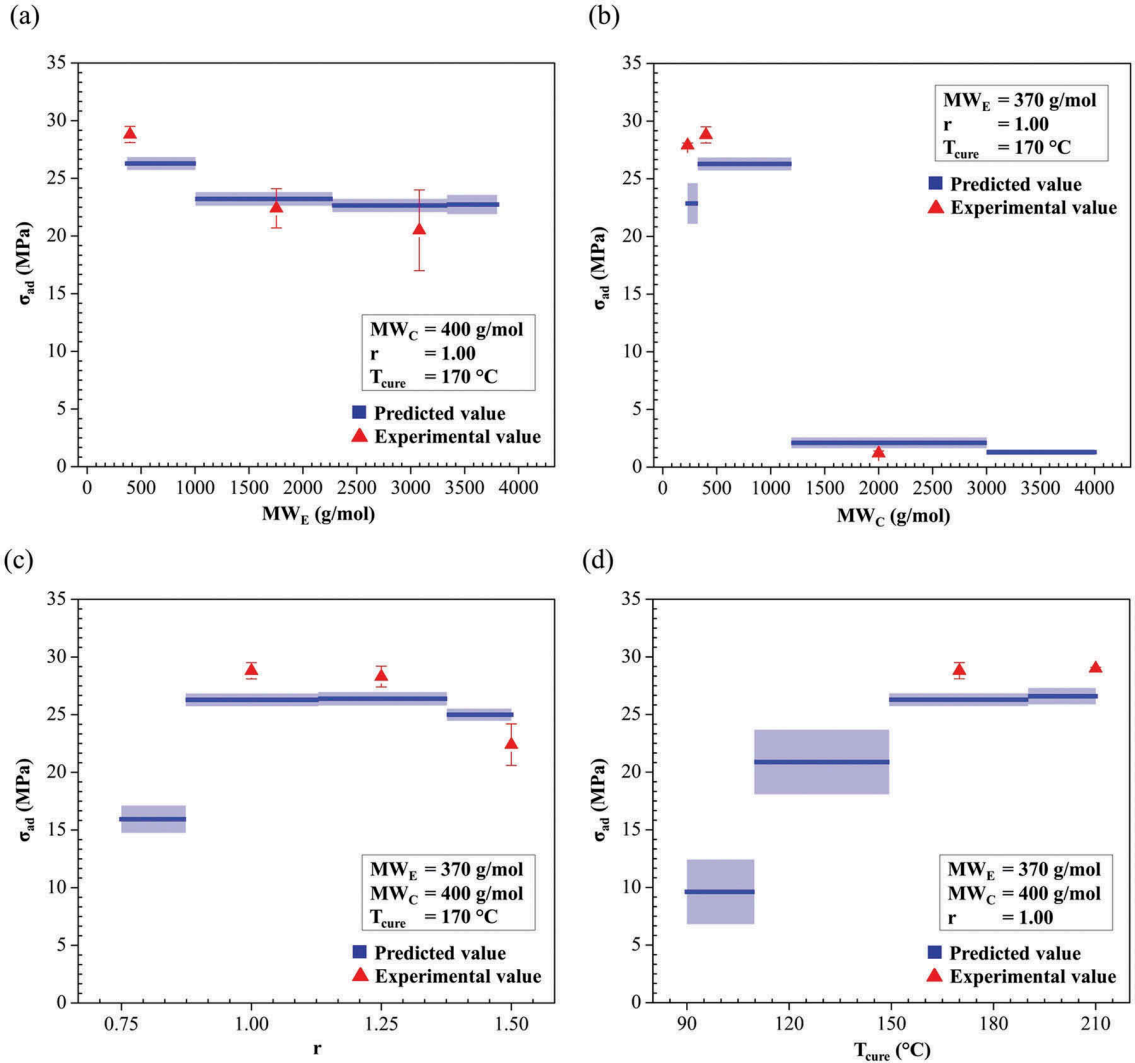
Predicted adhesive joint strength σ_ad_ (MPa) as a function of (a) molecular weight of epoxy resin MW_E_ (g/mol), (b) molecular weight of epoxy resin MW_C_ (g/mol), (c) amine-to-epoxide ratio r and (d) curing temperature T_cure_ (°C). The predicted σ_ad_ was calculated by averaging the predictions over the 47 folds of cross-validation. The blue line consisted of the predicted values of σ_ad_ (blue). Triangles represent experimental results (red).

We explore the influence of epoxy network structure on σ_ad_ of the joints through the developed ML model (Figure 9). The epoxy network structure was altered by varying the MW_E_, MW_C_, r and T_cure_ used to crosslink the adhesives. The predicted σ_ad_ were calculated by averaging the predictions over the 47 folds of cross-validation and their standard deviations are shown. The plots show a step change in the value of predicted σ_ad_. This step change corresponds to the decision-tree formation process in Gradient boosting within limited discrete input values. The experimental σ_ad_ values were plotted with their standard deviations. Although the bulk properties of various epoxy network structures have been studied extensively and reported previously [11], no comprehensive study focuses on their adhesive joint property, which is related more closely to the practical application of epoxy resin.

As shown in Figure 9(a), the σ_ad_ decreased slightly (i.e. less than 5 MPa) with an increase in MW_E_. This slight decrease of σ_ad_ for a high-MW epoxy resin most likely originates from an increased epoxy-resin viscosity. Because a higher MW_E_ possesses a higher viscosity, it is observed in the experiment that an adhesive that is prepared from a solid-type epoxy resin (MW_E_ = 1650, 2900 and 3800 g/mol) cannot spread well on the substrates, which results in a lowered adhesion strength between the adhesive and the substrates.

In the case of a curing agent, the σ_ad_ first increases with an increasing MW_C_, reaches a maximum of ~26 MPa at ~380–1200 g/mol, and then decreases sharply to less than 5 MPa (Figure 9(b)). The increase in σ_ad_ could be attributed to an enhanced flexibility within the crosslinked epoxy-amine network when the amine chain length is increased [13]. However, at a higher MW_C_ (> 1200 g/mol), the adhesive is too flexible to resist a high applied force, which results in a low σ_ad_. As observed in the experiment, the adhesives that were prepared with a MW_C_ above 2000 g/mol are extremely soft, which implies a much lower adhesive elastic modulus and tensile strength. This result is consistent with previous studies in which the elastic modulus of cured epoxies was reduced significantly from 2 GPa to 1.9 MPa when the molecular weight of Jeffamine was increased from 400 to 2000 g/mol [32,33].

For the amine-to-epoxide ratio effect, σ_ad_ increases first then it reaches a maximum, and then decreases slightly with an increase in r (Figure 9(c)). The high σ_ad_ from ~0.87 to 1.37 is attributed to the appropriate balance between flexibility and strength of adhesives [14,34].

The σ_ad_ increased gradually as T_cure_ increased and appears to be almost constant for a T_cure_ of 150–210°C (Figure 9(d)). Fully cured adhesives were obtained at a T_cure_ of 150–210°C because there is no significant difference in σ_ad_ and because of the physical appearance in this range. The low σ_ad_ region at a low T_cure_ between 90 and 150°C may indicate incomplete curing because the incomplete network structures of a partially cured adhesive result in a remarkably lower elastic modulus [35]. The experimental evidence shows that an adhesive cured at 90°C is relatively soft and/or the resin component remains liquid (uncured) compared with that cured at 170–210°C.

## 4. Conclusions

The design of experimental techniques combined with an active learning pipeline and Bayesian optimization was proposed to predict and optimize the adhesive joint strength (σ_ad_) of an epoxy-amine adhesive comprised of bisphenol A-based epoxy resin and amine-terminated poly(propylene glycol) curing agent with various molecular weights (MW_E_, MW_C_), mixing ratios (r) and curing temperatures (T_cure_). From an initial dataset of only 32 measured σ_ad_ with related epoxy-amine mixture preparation parameters {MW_E_, MW_C_, r, T_cure_}, our active leaning pipeline was able to propose preferred experimental conditions to build a predictive Gradient boosting model of σ_ad_ with an experimental-like error level, and to maximize the likelihood to design epoxy-amine adhesives with a high σ_ad_, along three cycles of active learning. An extremely high σ_ad_ of 35.8 ± 1.1 MPa was achieved using the experimental conditions that were refined by Bayesian optimization. Because the prediction model was built using a very small dataset (e.g. < 50 samples), and the efficiency of prediction was reasonably high (e.g. R^2^ > 0.8), our proposed approach is foreseen to reduce materials design and development time and cost, especially for which experimental datasets are rare.

Our predictive model also provides a physical understanding of adhesive systems over a wide range of parameters for preparation. A quantitative analysis indicates that high-strength adhesives require a MW_C_ of ~380–1200 g/mol, an r of ~0.87–1.37 and a T_cure_ above 150°C. However, a MW_E_ of 370–3800 g/mol has a slight effect on σ_ad_. Qualitatively, we emphasize that: (i) a balance between flexibility and strength of adhesives (by adjusting MW_C_, r) influences σ_ad_ significantly, (ii) a complete curing (high T_cure_) is compulsory to obtain a high σ_ad_ and (iii) an increase in epoxy viscosity (MW_E_) degrades the adhesive–substrate adhesion.

Future work on this topic should target multiple-objective optimization of an adhesive (e.g. adhesive joint strength, glass transition temperature and chemical resistance). Other molecular weights or epoxy resin and curing agent types can be added to the dataset to increase the design freedom of advanced high-strength adhesives. From an experimental perspective, structural and mechanical characterizations (e.g. crosslink density, dynamic mechanical analysis and fracture morphology) of the extremely high-strength adhesive achieved in this study are essential and will be conducted to elucidate the source of the exceptional properties, to guide experimentalists in the design of an epoxy-amine system for adhesive-bonding applications.
