# Supplementary material for: Prediction and optimization of epoxy adhesive strength from a small dataset through active learning
Source: Sci Technol Adv Mater. 2019 Oct 2;20(1):1010–21. doi: 10.1080/14686996.2019.1673670 (PMC6818118; doi:10.1080/14686996.2019.1673670)
Supplement: Supplemental Material [file TSTA_A_1673670_SM4906.pdf]

Supplemental material

Prediction and optimization of epoxy adhesive strength from a small dataset through active learning

Sirawit Pruksawan[a,b], Guillaume Lambard[c]*, Sadaki Samitsu[a], Keitaro Sodeyama[c] and Masanobu Naito[a,b,d]*

[a]*Data-driven Polymer Design Group, Research and Services Division of Materials Data and Integrated System (MaDIS), National Institute for Materials Science (NIMS), Tsukuba, Japan;* [b]*Program in Materials Science and Engineering, Graduate School of Pure and Applied Sciences, University of Tsukuba, Tsukuba, Japan;* [c]*Energy Materials Design Group, Research and Services Division of Materials Data and Integrated System (MaDIS), National Institute for Materials Science (NIMS), Tsukuba, Japan;* [d]*Department of Advanced Materials Science, Graduate School of Frontier Sciences, The University of Tokyo, Kashiwa, Japan*

* Corresponding author: LAMBARD.Guillaume@nims.go.jp, NAITO.Masanobu@nims.go.jp

Information Classification: General

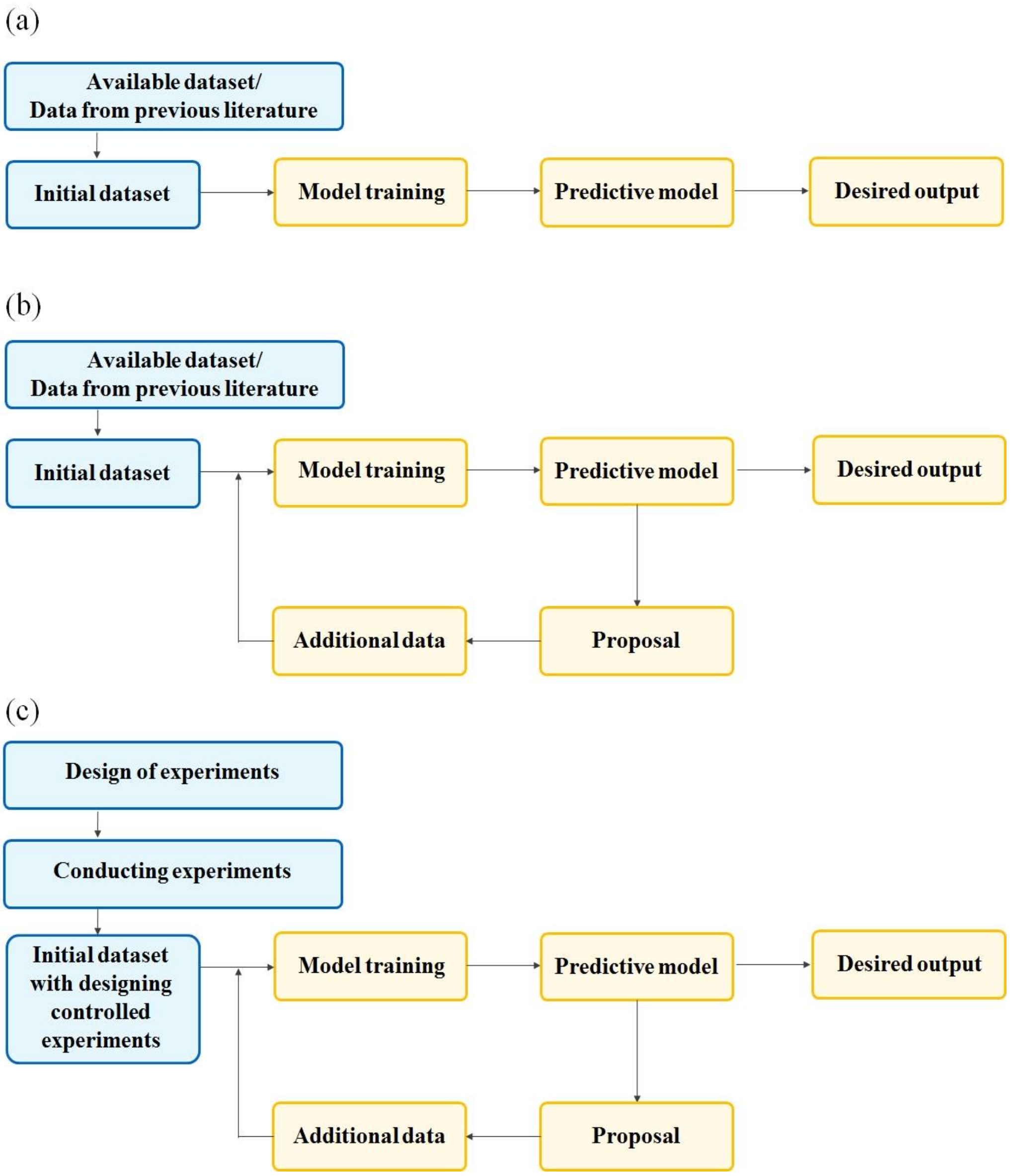

Figure S1. Typical workflow of a supervised machine learning task: (a) type A — conventional machine learning, (b) type B — active learning, (c) type C — active learning with designing controlled initial dataset.

Figure S1 shows three different types of supervised machine learning workflow: type A is the conventional machine learning, type B is active learning, and type C is our proposed approach — active learning with designing controlled initial dataset. In conventional machine learning (type A), an initial dataset collected from various sources

Information Classification: General

is used to train a predictive model, and the obtained model is applied for further prediction or optimization. Active learning (type B) is another strategy for machine learning in which the learning algorithm is fine-tuned by incorporating the new data point in its dataset. This type of iterative process is repeated cycle after cycle until a preliminary goal of a sufficiently high accuracy of the machine learning model is reached. The iterative process in active learning can improve the accuracy of the predictive model. In some case, an additional experimental data may be required to complete an initial dataset. Our proposed approach in this study (type C) is based on active learning; however, an initial dataset is constructed with designing controlled experiments and no any data from previous literature is required. Furthermore, the initial dataset obtained from experimental conditions suggested by design of experiment techniques allows us to attain a highly distributed dataset (Table S2), which is very beneficial for generating an accurate model.

Information Classification: General

Table S1. Summary of previous research work on machine learning-based experimental design in materials science. Workflow type refers to Figure S1.

Material	Target property	Feature data	Workflow type	Reference
$BaTiO_3$-based piezoelectrics	Large electrostrains	Data from previous literatures	B	[1]
Ferroelectric perovskites	High ferroelectric Curie temperature	Data from previous literatures	B	[2]
$BaTiO_3$-based ceramics	Large energy storage	Data from previous literatures	B	[3]
NiTi-based shape memory alloys	Low thermal hysteresis	Data from previous literatures	B	[4]
Metallic glasses	High glass-forming ability	Data from previous literatures	B	[5]
Epoxy adhesives	High adhesive joint strength	Data from chemical product information and process parameters	C	Our study

Information Classification: General

Table S2. Experimental results of adhesive joint strength σ_{ad} (MPa) of samples prepared under various conditions (initial dataset, dataset size n_s = 32 samples). Variable parameters include molecular weight of epoxy resin MW_E (g/mol), molecular weight of curing agent MW_C (g/mol) and amine-to-epoxide ratio r and curing temperature T_{cure} (°C).

No	Variable parameter				Measured σ_{ad} (MPa)	Appearance feature of adhesives (if any)
	MW_E (g/mol)	MW_C (g/mol)	r	T_{cure} (°C)		
1	370	230	0.75	90	8.3 ± 1.8	remaining uncured resin
2	370	400	1.00	170	28.8 ± 0.7	
3	370	2000	1.25	210	1.5 ± 0.1	Soft
4	370	4000	1.50	130	0.0 ± 0.0	soft; no adhesion to substrates
5	1650	230	1.00	130	18.0 ± 0.3	
6	1650	400	0.75	210	14.6 ± 1.0	orange-colored
7	1650	2000	1.50	170	3.3 ± 0.7	soft; sticky
8	1650	4000	1.25	90	2.0 ± 0.1	soft; remaining uncured resin
9	2900	230	1.25	170	17.7 ± 0.3	
10	2900	400	1.50	90	5.8 ± 0.5	
11	2900	2000	0.75	130	5.7 ± 0.3	
12	2900	4000	1.00	210	4.4 ± 0.5	soft; orange-colored
13	3800	230	1.50	210	15.3 ± 2.5	orange-colored
14	3800	400	1.25	130	10.4 ± 0.1	
15	3800	2000	1.00	90	1.2 ± 0.0	non-uniform; sticky
16	3800	4000	0.75	170	4.0 ± 0.1	
17	370	230	1.50	130	31.9 ± 0.1	
18	370	400	0.75	90	2.8 ± 1.5	remaining uncured resin
19	370	2000	1.00	170	1.2 ± 0.2	soft; sticky
20	370	4000	1.25	210	0.6 ± 0.0	soft; no adhesion to substrates
21	1650	230	1.25	90	9.9 ± 0.7	
22	1650	400	1.00	130	18.9 ± 0.8	
23	1650	2000	0.75	210	5.9 ± 0.7	soft; orange-colored
24	1650	4000	1.50	170	1.4 ± 0.5	soft; no adhesion to substrates
25	2900	230	1.00	210	23.1 ± 1.4	orange-colored
26	2900	400	1.25	170	24.6 ± 0.5	
27	2900	2000	1.50	90	4.4 ± 0.1	soft; remaining uncured resin
28	2900	4000	0.75	130	2.0 ± 0.6	
29	3800	230	0.75	170	15.5 ± 0.2	
30	3800	400	1.50	210	28.9 ± 0.6	orange-colored
31	3800	2000	1.25	130	13.5 ± 0.7	soft; sticky
32	3800	4000	1.00	90	0.0 ± 0.0	soft; no adhesion to substrates

Information Classification: General

Table S3. Experimental results of adhesive joint strength σ_{ad} (MPa) of samples prepared by active learning proposals (15 samples). Variable parameters include molecular weight of epoxy resin MW_E (g/mol), molecular weight of curing agent MW_C (g/mol), amine-to-epoxide ratio r and curing temperature T_{cure} (°C).

No	Variable parameter				Measured σ_{ad} (MPa)	Appearance feature of adhesives (if any)
	MW_E (g/mol)	MW_C (g/mol)	r	T_{cure} (°C)		
33	2900	400	1.00	210	24.0 ± 1.1	
34	3800	400	1.00	210	21.2 ± 1.2	
35	370	400	1.00	210	29.0 ± 0.1	orange-colored
36	1650	400	1.00	170	22.4 ± 1.7	
37	1650	400	1.00	210	27.3 ± 1.6	
38	370	400	1.25	210	27.8 ± 0.5	dark-colored
39	370	400	1.25	170	28.3 ± 0.9	
40	370	400	1.50	210	23.1 ± 0.4	dark-colored
41	370	400	1.50	170	22.4 ± 1.8	
42	1650	400	1.25	210	24.6 ± 0.0	orange-colored
43	2900	400	1.00	170	20.5 ± 3.5	
44	370	230	1.00	210	24.6 ± 2.0	dark-colored
45	370	230	1.00	170	27.9 ± 0.2	
46	1650	400	1.25	170	23.5 ± 1.0	
47	2900	400	1.25	210	25.7 ± 0.9	orange-colored

Information Classification: General

Table S4. Hyperparameters used for ML models. Default values of hyperparameters are used if no value is specified.

Model	Hyperparameter
Figure 5a	max_depth = 5, gamma = 3.3
Figure 5b	max_depth = 3, n_estimators = 100, random_state = 2
Figure 5c	alpha = 0.1
Table 2/Figure 6 – initial dataset	max_depth = 5, gamma = 3.30, learning_rate = 0.1
Table 2/Figure 6 – cycle 1	max_depth = 4, gamma = 2.70, learning_rate = 0.1
Table 2/Figure 6 – cycle 2	max_depth = 4, gamma = 1.80, learning_rate = 0.1
Table 2/Figure 6 – cycle 3	max_depth = 4, gamma = 2.86, learning_rate = 0.4

Information Classification: General

References

1. Yuan R LZ, Balachandran PV, Xue D, Zhou Y, Ding X, Sun J, Xue D, Lookman T. Accelerated Discovery of Large Electrostrains in $BaTiO_3$-based Piezoelectrics using Active Learning. Adv Mater.30(7):1702884.
2. Balachandran PV, Kowalski B, Sehirlioglu A, et al. Experimental Search for High-temperature Ferroelectric Perovskites Guided by Two-step Machine Learning. Nat Commun. 2018;9:1668.
3. Yuan R, Tian Y, Xue D, et al. Accelerated Search for $BaTiO_3$-based Ceramics with Large Energy Storage at Low Fields using Machine Learning and Experimental Design. Adv Sci. 2019:1901395.
4. Xue D, Balachandran PV, Hogden J, et al. Accelerated Search for Materials with Targeted Properties by Adaptive Design. Nat Commun. 2016;7:11241.
5. Ren F, Ward L, Williams T, et al. Accelerated Discovery of Metallic Glasses through Iteration of Machine Learning and High-throughput Experiments. Sci Adv. 2018;4(4):eaaq1566.

Information Classification: General
